# Progress in reprogramming failed graft-versus-leukemia immunity in acute myeloid leukemia relapse after allogeneic transplantation

**DOI:** 10.3389/fimmu.2026.1910630

**Published:** 2026-09-03

**Authors:** Naif I. AlJohani, Ahlam Almasari, Zayed Alzahrani, Nahla Shihata, Binyam Usman, Bassim Albeirouti

**Affiliations:** Oncology Department, King Faisal Specialist Hospital and Research Centre, Jeddah, Saudi Arabia

**Keywords:** acute myeloid leukemia, allogeneic stem cell transplantation, CAR-NK, CAR-T, CD83, HLA-directed therapy, posttransplant relapse

## Abstract

Relapse of acute myeloid leukemia (AML) after allogeneic hematopoietic stem cell transplantation (allo-HSCT) affects 40–50% of recipients and remains the leading cause of posttransplant mortality; median overall survival is approximately 5 months and 1-year survival is below 20%. Conventional salvage—chemotherapy, immunosuppression withdrawal, donor lymphocyte infusion (DLI), hypomethylating agents, targeted agents, and second transplantation—benefits a minority and has improved only incrementally over two decades. Relapse in this setting reflects leukemia that has survived an engrafted allogeneic immune system. Documented escape mechanisms include genomic loss of the mismatched human leukocyte antigen (HLA) haplotype, epigenetic downregulation of class II HLA molecules, checkpoint ligand upregulation, microenvironmental suppression, and clonal evolution—each calling for targeted redirection of the graft-versus-leukemia (GVL) response rather than further nonspecific cytotoxicity. We propose a GVL Failure Framework that organizes posttransplant relapse into three biologically defined categories of immune escape, aligns matched cellular therapies with each, and overlays a separate clinical transplant-eligibility axis that sets the therapeutic goal. Several cellular strategies have matured: autologous and donor-derived chimeric antigen receptor T (CAR-T) cells targeting CD33, CD123, C-type lectin-like molecule 1 (CLL-1)/CD371, and CD117; armored CAR-T products secreting interleukin-18; CD83-directed CAR-T cells targeting blasts and alloreactive T cells simultaneously; HLA-DRB1-directed CAR-T and chimeric antigen receptor natural killer (CAR-NK) cells exploiting donor-recipient mismatch for leukemia specificity; and off-the-shelf CAR-NK platforms reprogramming innate rather than adaptive allogeneic immunity. We integrate the mechanistic rationale and early clinical evidence for each approach, propose a four-pathway treatment algorithm built around relapse-clone HLA typing before DLI, and outline the gaps that must close before cellular therapy enters standard practice.

## 1 Introduction

For patients with intermediate- or high-risk acute myeloid leukemia (AML), allogeneic hematopoietic stem cell transplantation (allo-HSCT) remains the only consistently curative therapy. Despite advances reducing 2-year non-relapse mortality below 10%, relapse occurs in 40–50% of patients and remains the leading cause of treatment failure; median overall survival is measured in months and 2-year survival is below 15% (1, 2). Donor lymphocyte infusion (DLI), hypomethylating agents with or without venetoclax, and molecularly targeted agents add incremental value, but no component of the current toolkit reliably produces durable remission in more than a minority of patients (3).

Relapse after transplantation is not simply a return to refractory leukemia. Leukemia has survived an engrafted allogeneic immune system and carries the imprint of that selection pressure. Reported escape mechanisms include genomic loss of the mismatched human leukocyte antigen (HLA) haplotype (4, 5); epigenetic downregulation of class II HLA molecules (6, 7); checkpoint ligand upregulation; microenvironmental suppression; and clonal evolution. Each mechanism may help explain why nonspecific rekindling of graft-versus-leukemia (GVL) by DLI succeeds in only a few patients.

This review proposes a unifying framework—the GVL Failure Framework—that organizes posttransplant relapse biology into three categories of immune escape, overlaid by a clinical transplant-eligibility axis, and maps each cellular modality to the failure mode it addresses. We then examine why the posttransplant setting is paradoxically favorable for chimeric antigen receptor T-cell (CAR-T) therapy, survey conventional salvage benchmarks and five cellular platform classes that have matured since 2024, propose a clinical algorithm, and discuss whether cellular therapy may eventually replace second transplantation.

## 2 The GVL failure framework: three categories of immune escape and a clinical eligibility axis

**Figure 1:**
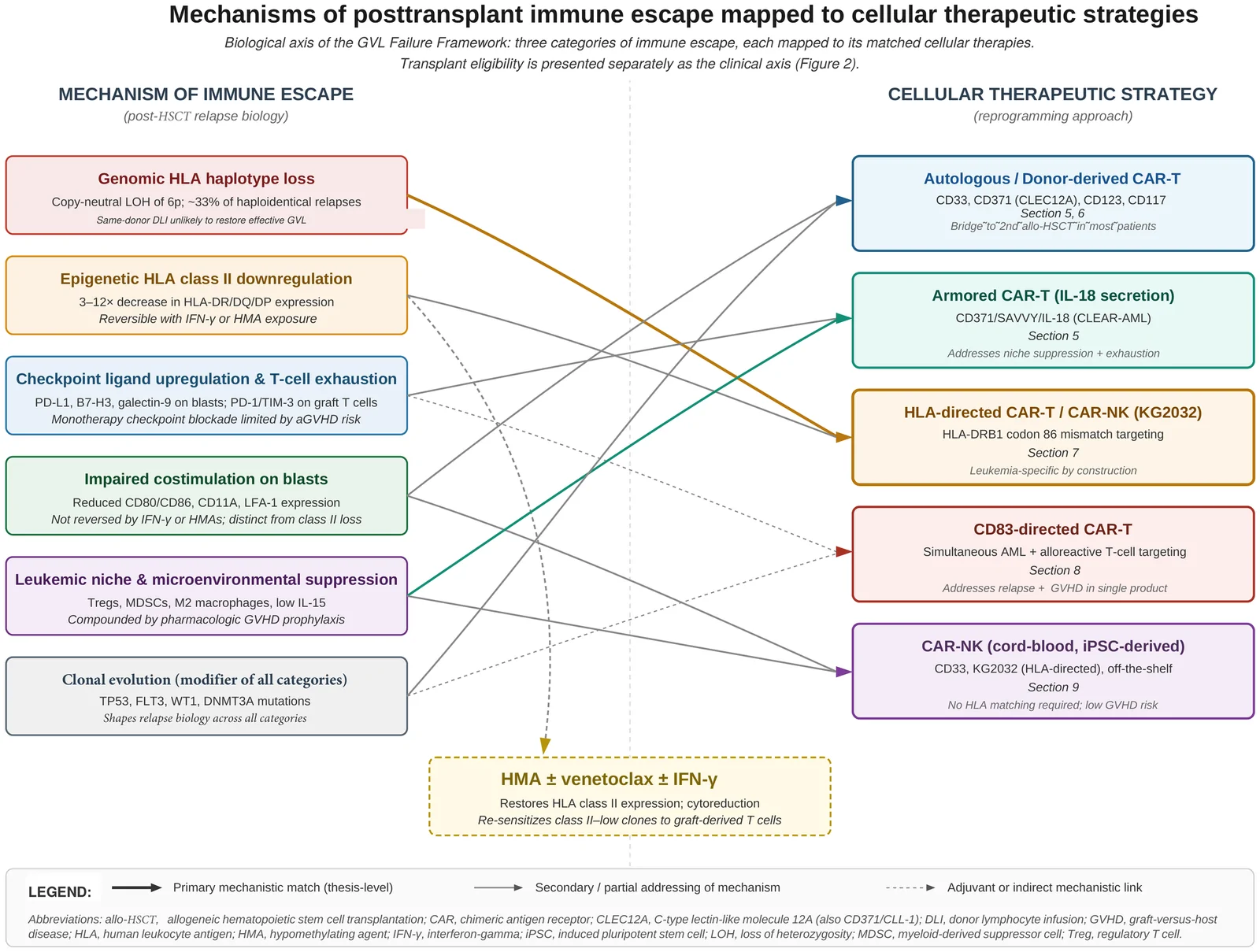
Mechanisms of posttransplant immune escape mapped to cellular therapeutic strategies. Each cellular modality is positioned against the putative failure mode of the graft-versus-leukemia response it is best suited to address; the mechanism-to-therapy alignment shown is a hypothesized optimal matching rather than an established causal relationship. Mechanisms of immune escape (left) include genomic HLA haplotype loss, epigenetic HLA class II downregulation, checkpoint ligand upregulation with graft T-cell exhaustion, impaired costimulation on blasts, leukemic niche and microenvironmental suppression, and clonal evolution. Matched cellular therapeutic strategies (right) include autologous and donor-derived CAR-T cells targeting CD33, CD371, CD123, or CD117; armored CAR-T constructs secreting interleukin-18; HLA-directed CAR-T and CAR-NK platforms (KG2032) exploiting donor–recipient HLA-DRB1 mismatch; CD83-directed CAR-T cells simultaneously targeting blasts and alloreactive T cells; and off-the-shelf CAR-NK cells derived from cord blood or induced pluripotent stem cells. Solid arrows denote the primary mechanistic match; thin arrows denote secondary or partial coverage; dashed arrows denote adjuvant or indirect mechanistic links. The bottom box highlights hypomethylating agents with or without venetoclax and interferon-gamma as adjuvants that restore HLA class II expression. Abbreviations: allo-HSCT, allogeneic hematopoietic stem cell transplantation; CAR, chimeric antigen receptor; CLEC12A, C-type lectin-like molecule 12A (also CD371/CLL-1); DLI, donor lymphocyte infusion; GVHD, graft-versus-host disease; HLA, human leukocyte antigen; HMA, hypomethylating agent; IFN-γ, interferon-gamma; iPSC, induced pluripotent stem cell; LOH, loss of heterozygosity; MDSC, myeloid-derived suppressor cell; Treg, regulatory T cell.

Posttransplant AML relapse is biologically distinct from chemotherapy-only relapse: selection pressure from an engrafted allogeneic immune system drives immune-evasion phenotypes largely absent pretransplant. We organize this biology along two orthogonal axes. The first is a biological classification of immune escape into three categories that capture the spectrum of posttransplant relapse biology and dictate which cellular target and strategy are required (**Figure 1**). The second is a clinical axis—transplant eligibility—that is not itself an escape mechanism but determines the therapeutic goal, whether cytoreductive bridging or definitive transplant-free therapy, and is developed in the treatment algorithm (Section 10). Separating the two prevents conflation of relapse biology with clinical decision-making.

Category 1—Antigen-loss escape. Genomic loss of the mismatched HLA haplotype was first described by Vago et al. (4) after haploidentical transplantation with donor T-cell infusion. The mechanism is copy-neutral loss of heterozygosity on chromosome 6p, eliminating patient-specific HLA alleles while retaining overall HLA expression (8). The relapsed clone becomes invisible to donor T cells without triggering a natural killer (NK) response because class I expression is preserved. Antigen loss occurs in approximately one-third of relapses after haploidentical transplantation and at lower frequencies after other HLA-mismatched platforms (5, 9, 10). A large multicenter analysis further established that HLA loss occurs across donor types—not only after haploidentical transplantation—and that systematic characterization of the relapse clone’s HLA status, together with donor selection, is central to identifying the appropriate targeted strategy for each patient (11). Same-donor DLI lacks a biological target here.

Category 2—Antigen-silencing escape. Profound downregulation of HLA class II without genomic alteration defines this category. Christopher et al. (6) showed 3- to 12-fold lower HLA-DR/DP/DQ expression at relapse than at presentation, with blasts unable to stimulate allogeneic CD4+ T cells. Downregulation is epigenetic and reversible: interferon-gamma restores class II expression within 72 hours *in vitro*, and hypomethylating agents produce an analogous effect, providing a rationale for azacitidine and decitabine activity independent of cytotoxicity (7, 12). Beyond re-sensitizing clones to the endogenous graft, this epigenetic re-expression of HLA class II offers a neoadjuvant strategy to raise target-epitope density before delivery of HLA-directed or microenvironment-armored cellular platforms. Reduced CD80, CD86, CD11A, and lymphocyte function-associated antigen 1 (LFA-1) expression compounds this by impairing the second signal for T-cell activation.

Category 3—Immune-exhaustion escape. The T-cell compartment is dysfunctional: leukemic blasts express elevated programmed death-ligand 1 (PD-L1), B7 homolog 3 (B7-H3), and galectin-9, while graft-derived T cells express increased programmed cell death protein 1 (PD-1), T-cell immunoglobulin and mucin domain-containing protein 3 (TIM-3), and lymphocyte activation gene 3 (LAG-3). Checkpoint inhibition monotherapy shows only modest activity and carries a risk of acute graft-versus-host disease (GVHD) (13). The microenvironment compounds this through enriched regulatory T cells, myeloid-derived suppressor cells, and M2-polarized macrophages; low serum interleukin-15 (IL-15) levels early posttransplant are associated with a higher relapse risk (1). Metabolic rewiring of the microenvironment contributes further: donor T-cell antileukemic function is constrained by nutrient competition and altered metabolism, and metabolic reprogramming of donor T cells has been shown to enhance graft-versus-leukemia activity in preclinical and translational models (14). This category provides the rationale for armored CAR constructs and CD83-directed strategies.

**Figure 2:**
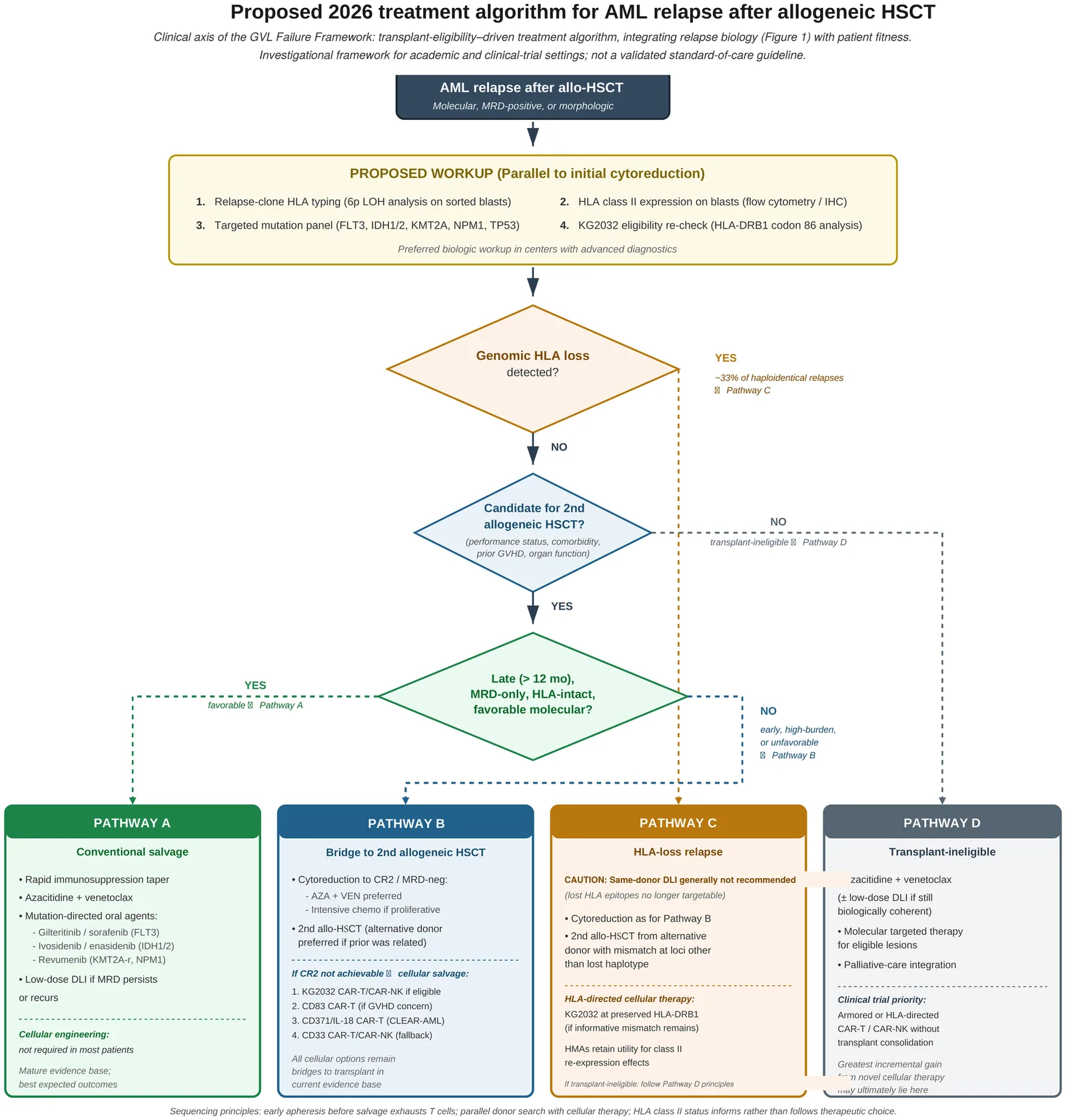
Proposed 2026 treatment algorithm for AML relapse after allogeneic HSCT. This is a proposed investigational framework intended for academic and clinical-trial settings, not a validated treatment guideline. Pathway assignment is driven by relapse biology (HLA-loss status, HLA class II expression, molecular profile) and patient fitness. A proposed workup performed in parallel with initial cytoreduction includes relapse-clone HLA typing using 6p loss-of-heterozygosity analysis on sorted blasts, HLA class II expression by flow cytometry or immunohistochemistry, a targeted mutation panel (FLT3, IDH1/2, KMT2A, NPM1, TP53), and KG2032 eligibility re-check using HLA-DRB1 codon 86 analysis. The first decision point is whether genomic HLA loss is detected; an affirmative finding (approximately one-third of haploidentical relapses) routes patients to Pathway C, in which same-donor lymphocyte infusion is contraindicated and HLA-directed cellular therapy at preserved alleles becomes the central option. In the absence of HLA loss, candidacy for a second allogeneic HSCT is assessed; transplant-ineligible patients are routed to Pathway D. Transplant-eligible patients are further stratified by late versus early relapse, mutational risk, and HLA integrity: favorable cases enter Pathway A (conventional salvage); unfavorable or early high-burden cases enter Pathway B (cytoreductive bridge to second allogeneic HSCT, with cellular salvage as a bridge if CR2 is not achievable). Sequencing principles include early apheresis before salvage exhausts T cells, conducting the donor search in parallel with cellular therapy, and use of HLA class II status to inform rather than follow therapeutic choice.

The clinical eligibility axis. Orthogonal to these three biological categories is a clinical dimension that shapes therapeutic intent independently of escape mechanism: whether the patient remains a candidate for further intensive immunologic reconstitution, in particular a second allogeneic transplant. A substantial subset of relapsing patients is transplant-ineligible owing to comorbidity, prior severe GVHD, or organ dysfunction. For transplant-eligible patients, cellular therapy generally serves as a cytoreductive bridge to transplant; for transplant-ineligible patients, the same biological category instead demands a construct that can work without subsequent transplantation, making durability rather than bridging the priority. Because this is a clinical rather than mechanistic distinction, it is represented not as a fourth escape category but as the eligibility axis that drives pathway assignment in the treatment algorithm (Section 10; **Figure 2**).

Clonal evolution operates across all three categories and shapes rather than initiates immune escape. Genetic instability at relapse is nonetheless substantial: a recent multicenter analysis found acquisition or loss of mutations in roughly two-thirds of patients relapsing after transplantation—most often involving FLT3-ITD, NRAS, and KRAS, with founding lesions such as DNMT3A typically retained—and identified constant, linear, branching, or parallel evolutionary trajectories (15). This dynamic selection under therapeutic and immune pressure means posttransplant relapse is not simply re-emergence of the original clone; nonetheless, its distinguishing feature relative to post-chemotherapy relapse is immune escape rather than the mutational spectrum itself, and reversing it requires restoring an immune response, not only eliminating a new genetic lesion. Effective reprogramming therefore requires one of three biologically matched strategies—an antigenic target invariant under allogeneic selection (Category 1), combination with agents reversing the specific escape mechanism (Category 2), or co-engineering to resist suppression (Category 3)—deployed as a bridge or as definitive therapy according to the patient’s position on the transplant-eligibility axis.

These mechanisms also bear on the timing and detectability of relapse. Immune escape, HLA loss, and clonal shifts develop progressively under selective pressure and are frequently detectable at the molecular level before overt hematologic relapse, so longitudinal molecular tracking of the relapse clone can enable earlier and potentially mechanism-specific intervention (16). Relapse timing is itself prognostic—relapse within the first 6 months carries markedly worse outcomes (15)—which argues for intensified molecular monitoring during that window and for prioritizing measurable residual disease targets among mutations that persist rather than those lost at relapse.

## 3 Why CAR-T works better after transplant than before

A central paradox of CAR-T therapy for AML is that the disease setting in which it has performed worst—myeloid disease before transplant—is precisely the setting in which engineering challenges are greatest. Myeloid antigens are shared with hematopoietic progenitors, and on-target/off-tumor myelosuppression is difficult to reconcile with sustained responses. The posttransplant compartment offers four solutions to constraints that cannot be resolved in pretransplant AML.

T-cell starting material. In patients with stable donor chimerism, the peripheral T-cell compartment is predominantly donor-derived, even when the cells are collected from the patient. Donor-derived effectors—harvested from the engrafted recipient or from a fresh donor draw—are less heavily pretreated than autologous T cells from chemotherapy-only AML.

Marrow backup. On-target myelotoxicity is the most consistent limitation of myeloid-antigen CAR-T. After transplantation, the donor graft serves as biological insurance against prolonged aplasia, and clustered regularly interspaced short palindromic repeats (CRISPR)-edited grafts can be engineered to resist the targeted antigen entirely (Section 6).

Mismatch-derived specificity. Pretransplant AML offers no leukemia-restricted surface antigen that is not also expressed on normal hematopoietic progenitors. The posttransplant context introduces a new source of specificity: HLA disparities between donor and recipient that the leukemia retains but the engrafted graft does not (Section 7).

Effector support. Healthy donor hematopoiesis provides a continuous supply of NK cells and homeostatic cytokine support that armored CAR-T and chimeric antigen receptor natural killer (CAR-NK) constructs leverage. The posttransplant setting is not where CAR-T struggles despite immunological complexity; it is where CAR-T should succeed because of it.

## 4 Conventional salvage: benchmarks and their limits

Conventional management rests on four pillars: tapering immunosuppression, cytoreductive salvage, DLI, and second transplantation for selected fit patients, with molecularly targeted agents integrated across these approaches. None reliably achieves durable remission in more than a minority of patients (2).

Rapid taper of calcineurin inhibitors at the first sign of relapse remains the initial maneuver but produces durable remission in only a small fraction of patients. Therapeutic DLI, typically preceded by cytoreduction, achieves long-term remission in approximately 15–25% of patients, with higher rates in late relapse beyond 6 months, low disease burden, and favorable cytogenetics (2). DLI is limited by the risk of acute and chronic GVHD (30–50%), the requirement for a cooperative donor (unavailable for cord blood recipients), and the absence of a biological rationale when relapse occurs through Category 1 antigen-loss. Routine HLA typing of the relapsed clone before DLI is an evidence-based prerequisite (4, 5), and this applies regardless of donor type rather than only after haploidentical transplantation: HLA loss is now documented across donor platforms, and characterizing it at relapse both identifies patients for whom same-donor DLI is futile and informs donor selection if a second transplant is pursued (11).

Azacitidine or decitabine combined with venetoclax substantially improves cytoreduction. A retrospective series reported complete response rates of approximately 40% with a meaningful proportion of measurable residual disease (MRD)-negative remissions (17, 18). The combination provides a biologically rational platform for HLA class II re-expression in Category 2 disease. Most series pair hypomethylating agent (HMA)–venetoclax therapy with DLI for sustained benefit; standalone responses are rarely durable beyond 1 year.

Targeted agents refine treatment for defined subsets. In FLT3-ITD AML, SORMAIN showed that sorafenib maintenance improved 2-year relapse-free survival from 53% to 85% (HR 0.39) (19). MORPHO showed a significant relapse-free survival benefit in patients with detectable posttransplant MRD (20). IDH1 and IDH2 inhibitors produce single-agent response rates of 20–30%. Revumenib, approved in 2024 for KMT2A-rearranged AML and in 2025 for NPM1-mutated relapsed/refractory AML, has extended durable responses to additional molecular subsets (21, 22). These agents address residual leukemic clones but not the breach of GVL control and are typically bridges to cellular salvage or second transplantation.

For fit patients achieving remission, second allo-HSCT remains the most intensive conventional option. European Society for Blood and Marrow Transplantation (EBMT) data on 1,540 transplantations document progressive improvement, with cumulative relapse incidence declining from 64% to ~51% (23, 24). Non-relapse mortality and GVHD incidence did not improve. Most patients are not candidates owing to comorbidities, prior GVHD, or organ toxicity. Across modalities, 2-year overall survival reaches 25–40% only in highly selected patients. This gap defines the role for cellular engineering (**Table 1**).

**Table 1:** Selected cellular therapies for acute myeloid leukemia relapse after allogeneic hematopoietic stem cell transplantation.

Target	Construct/trial	Cell source	Phase (n)	Response/key findings	Key safety (CRS; GVHD)	Status [Ref]
CD371 (CLEC12A)	CD371/SAVVY/IL-18 CLEAR-AML (NCT06017258)	Autologous (donor-origin post-HSCT)	1 (n=5; 3 post-HSCT)	3/3 post-HSCT patients achieved MLFS with MRD negativity; DLTs at 3 × 10^5^ CAR-T cells/kg (marrow aplasia and grade 4 CRS) → dose reduced to 3 × 10^4^ CAR-T cells/kg	At 3 × 10^5^ CAR-T cells/kg, DLTs were grade 4 CRS in one patient and prolonged cytopenias with marrow hypoplasia in one; no GVHD observed	Active (25)
CD33	Humanized CD33 CAR-T (Lin/Tong, Beijing Gobroad Boren)	Autologous or donor-derived	1 (n=12 post-HSCT)	CR 41.7% (5/12); CRS grade 1–2; no ICANS; 5/12 bridged to second allo-HSCT; 1 durable CMR without second HSCT	CRS in 10/12 patients (9 grade 1, 1 grade 2); no ICANS; no confirmed GVHD reported, although 4/12 had liver dysfunction possibly related to GVHD or CRS	Ongoing (26)
CD33	VCAR33 (lintuzumab scFv, CD28) VBP301 (NCT05984199)	Donor-derived (original 8/8 matched donor)	1/2 (n=15)	ORR 20% (2 CRi + 1 MRD clearance); CRS 93% (all < grade 3); ICANS 27% (1 ≥ grade 3); grade III acute GVHD 7%	CRS 93% (all <G3); ICANS 27% (1 ≥G3); grade III aGVHD 7%	Terminated (non-safety) (28)
CLL-1	Donor-derived CLL-1 CAR-T (Zhang et al., ChiCTR2000041054)	Donor-derived	1 (n=12 post-HSCT)	Activity across three dose levels (0.5–1.5 × 10^6^ cells/kg); severe neutropenia was a major barrier; bridging to second allo-HSCT or donor stem cell infusion extended survival	CRS in 12/12 patients (5 with grade ≥ 3); CRES in 3/12, including one grade 4 CRS/CRES-related death; severe pancytopenia in all patients; GVHD occurred after subsequent HSCT	Ongoing (31)
CD123	Donor-derived CD123 CAR-T (NCT03114670)	Donor-derived	1	Limited published data; on-target myelotoxicity remains a central challenge	Not reported	Status unknown
CD123	AVC-201: Allo-RevCAR01-T + R-TM123 adapter; RevSTAR-123 (NCT05949125)	Off-the-shelf allogeneic; CRISPR-edited universal-donor T cells (switchable)	1a (n=17)	Reversible “on/off” control via short-acting adapter; CRS 76% (6% ≥G3), no GVHD/ICANS, 1 DLT (G2 IEC-HS); dose-dependent depth at DL15 with CRs, MRD conversion, and MLFS incl. TP53-mutant AML; cohort not restricted to post-HSCT relapse	CRS 76% (6% ≥G3); no GVHD or ICANS; 1 DLT (G2 IEC-HS)	Phase 1b ongoing (32)
CD117	Donor-derived CD117 CAR-T (Zhejiang Univ, NCT07144020)	Donor-derived	1	LSC-enriched target; recently opened; no clinical results yet	Not yet reported	Recruiting
CD33	Trem-cel + gemtuzumab ozogamicin (VBP101)	CRISPR-edited CD33-KO HSC graft + CD33-ADC	1/2	30 received trem-cel; 19 received GO maintenance; all 30 achieved neutrophil engraftment by day 28; sustained CD33-negative donor hematopoiesis enabled repeat GO dosing to the RP2D of 2 mg/m² without prolonged high-grade cytopenias.	CRS not applicable; grade 2–4 acute GVHD 20% (grade 3–4, 7%); chronic GVHD 7%; GO tolerated to RP2D without prolonged high-grade cytopenias; 3 transplant-related deaths (renal failure, sepsis, SOS/VOD)	Final report; trial stopped early (33)
HLA-DRB1 (codon 86)	KG2032 CAR-T/CAR-NK (Ikeda/Hosen, Osaka)	Donor T cells (DRB1-negative for reactive allele) or cord-blood NK	Preclinical	Potent and specific anti-AML activity in xenograft; no overt toxicity in treated mice; graft-sparing by donor–recipient mismatch design; ~50% eligibility across mismatched donor pairs	Preclinical; no overt toxicity in xenografts	Clinical trials planned (35)
CD83	CD83 CAR-T (Roswell Park, NCT06871410)	Autologous (donor-origin post-HSCT)	1	Preclinical: AML killing + xenogeneic GVHD prevention/treatment + antiviral preservation + calcineurin-inhibitor-resistant; graft-sparing (CD83 absent on HSCs); first-in-human dosing initiated April 2026	Preclinical; no clinical safety data yet	Active (no clinical data yet) (37, 39)
CD33	Cord-blood CD33 CAR-NK (Huang et al.)	Cord-blood-derived NK	1 (n=10)	6/10 patients (60%) achieved MRD-negative CR at day 28; no ICANS; median PFS 71.5 days, median OS 137 days in the CR subgroup; only the patient bridged to allo-HSCT reached long-term remission	Grade 2 CRS in 1/10 patients; no ICANS or GVHD; grade 3–4 bone marrow suppression resolved within 1 month	Completed (42)
CD33	CD33 CAR-NK-92 (Tang et al.) First-in-man NK-92 CAR trial	NK-92 cell line	1 (n=3)	Safety-oriented; no sustained remissions; established the safety of the CAR-NK class	Well tolerated; safety-oriented (n=3)	Completed (43)

Selected clinical and preclinical programs referenced in the text, grouped by target antigen and cell source. Response and toxicity data reflect the most recent reports available at the time of writing and should be interpreted in the context of differing eligibility criteria, disease burden at enrollment, and follow-up duration across trials. ADC, antibody-drug conjugate; allo-HSCT, allogeneic hematopoietic stem cell transplantation; CAR, chimeric antigen receptor; CLL-1, C-type lectin-like molecule 1 (also CD371/CLEC12A); CMR, complete molecular remission; CR, complete remission; CRi, complete remission with incomplete count recovery; CRS, cytokine release syndrome; DLT, dose-limiting toxicity; GO, gemtuzumab ozogamicin; GVHD, graft-versus-host disease; HSC, hematopoietic stem cell; ICANS, immune effector cell–associated neurotoxicity syndrome; KO, knockout; LSC, leukemic stem cell; MLFS, morphologic leukemia-free state; MRD, measurable residual disease; NK, natural killer; ORR, overall response rate; OS, overall survival; PFS, progression-free survival; scFv, single-chain variable fragment.

Notes: “Autologous” in the posttransplant setting refers to apheresis from a patient with stable donor chimerism; the collected cells are predominantly donor-origin. “Donor-derived” refers to fresh apheresis from the original hematopoietic cell donor. Numbers in parentheses in the rightmost column refer to entries in the numbered reference list. Where no number appears, the trial is registered but has not yet generated a published primary report; the relevant registry identifier (NCT or ChiCTR) is listed in the Construct/Trial column.

## 5 Autologous CAR-T in the posttransplant patient

Feasibility of autologous CAR-T manufacturing has been demonstrated despite four challenges: lymphopenia from prior conditioning, functional exhaustion from chronic antigen exposure, the suppressive microenvironment, and the need to briefly hold pharmacologic GVHD prophylaxis around apheresis (25). In CLEAR-AML, autologous manufacturing succeeded in all five patients (median production time, 7 days). Similar feasibility was reported in a Chinese series of CD33- and C-type lectin-like molecule 1 (CLL-1)-directed products (26). Yield and T-cell fitness are maximized when apheresis is performed at the earliest sign of relapse.

The most informative recent experience comes from CLEAR-AML (NCT06017258), evaluating CD371/SAVVY/IL-18 CAR-T cells (25). The construct combines a fully human CD371-directed single-chain variable fragment (scFv), a mutated CD28 costimulatory domain engineered to limit exhaustion (SAVVY), and constitutive secretion of interleukin-18 (IL-18), which acts as both an autocrine stimulus for CAR-T expansion and an activator of endogenous NK cells. CD371 (CLEC12A, CLL-1) is expressed on AML blasts and leukemic stem cells but largely absent from hematopoietic stem cells. All three posttransplant patients achieved morphologic leukemia-free state with MRD negativity, and the median time to first response was 15 days. Dose-limiting toxicities at 3 × 10^5^ CAR-T cells/kg led to a dose reduction to 3 × 10^4^ CAR-T cells/kg. Single-cell transcriptomics showed expansion of cytotoxic CD8+ effectors with concomitant NK activation, consistent with IL-18–driven amplification of adaptive and innate compartments. Durability was limited: two responders required subsequent consolidation (both followed by relapse) and a third died of adenoviral reactivation. The construct produced rapid, mechanistically coherent clearance, but durability required consolidation.

CD33-directed autologous CAR-T cells have the longest clinical track record. Lin et al. (26) reported 12 patients with relapsed/refractory (R/R) AML after allo-HSCT who received humanized CD33 CAR-T cells at doses of 6.2 × 10^4^ to 6.15 × 10^5^ cells/kg: the complete remission rate was 41.7%, cytokine release syndrome (CRS) was largely grade 1–2, immune effector cell–associated neurotoxicity syndrome (ICANS) was absent, and 5 of 12 patients were successfully bridged to second transplantation. CLL-1-directed autologous constructs in Chinese series show encouraging response rates (27). Durable remission was achieved almost exclusively in patients who underwent consolidation with second transplantation. Dual-antigen constructs (CD33–CLL-1, trispecific CLL-1–CD33–NKG2D) have reported complete response rates of 70–80% in R/R AML cohorts (27), though post-HSCT data are limited.

Three limitations constrain the role of autologous CAR-T in post-HSCT AML relapse: donor-origin T cells may exhibit exhaustion limiting expansion and persistence; on-target myelotoxicity against shared myeloid antigens leads to prolonged cytopenias; and durable responses without consolidation remain rare. Autologous CAR-T is best viewed as a cytoreductive bridge to second transplantation.

## 6 Donor-derived CAR-T: reprogramming the graft itself

The central safety concern for donor-derived CAR-T is graft-versus-host disease, because infused cells retain endogenous T-cell receptors alongside the synthetic CAR. In practice, severe GVHD has been lower than initial models predicted, likely because lymphodepletion, expansion kinetics, and small absolute numbers of infused cells limit alloreactive engraftment. Across published donor-derived CAR-T series in post-HSCT AML, grade III or higher acute GVHD remains under 10% (28). T-cell receptor disruption approaches, including CRISPR-Cas9 T-cell receptor alpha constant (TRAC) knockout, zinc-finger nucleases, and transcription activator-like effector nucleases (TALENs), bring donor-derived products closer to off-the-shelf allogeneic products but have not consistently outperformed matched-donor unmodified CAR-T.

The most complete clinical evidence comes from VBP301 (NCT05984199), evaluating VCAR33—a lintuzumab-derived CD33-directed CAR with CD28 costimulation manufactured from each patient’s original 8/8 HLA-matched donor. Initial data were presented at ASH 2023 (29), with first-15-patient results published by Mushtaq et al. (28): seven patients with morphological disease and eight with measurable residual disease received VCAR33 after fludarabine-cyclophosphamide lymphodepletion. CRS occurred in 93% (all < grade 3), ICANS in 27% (one ≥ grade 3), and grade III acute GVHD in 7%. The overall response rate was 20%. The study was terminated before escalation to the third planned dose level for non-safety reasons. Efficacy at the tested doses did not exceed contemporaneous autologous CD33 CAR-T (30). The modest 20% response rate is best read not as a definitive failure of the CD33 target but as the consequence of a trial halted for strategic, non-safety reasons before the highest planned dose level was reached, so activity was assessed largely at potentially subtherapeutic doses; limited *in vivo* persistence of the donor-derived product—rather than documented antigen escape or inadequate lymphodepletion—is the most plausible contributor at those dose levels, although this interpretation remains inferential and the relative contributions of persistence, disease burden, and other biological factors were not established. Termination despite acceptable safety illustrates the structural, as much as biological, challenges of donor-derived CAR-T development in AML.

Donor-derived CLL-1 CAR-T cells were evaluated by Zhang et al. (31) in a Chinese single-center trial (ChiCTR2000041054): 12 of 14 patients received donor-derived CLL-1 CAR-T at three dose levels, and bridging to second transplantation substantially shortened cytopenia duration. Additional donor-derived programs include CD123 (NCT03114670) and a CD117 platform at Zhejiang University (NCT07144020). CD117, a leukemic stem cell–enriched marker, is biologically attractive but carries substantial myelotoxicity risk that donor-derived delivery alone does not resolve.

A conceptually different solution to the same myelotoxicity problem controls the activity of the CAR rather than the antigen or the graft. The switchable allogeneic platform AVC-201 pairs CRISPR-edited universal-donor T cells expressing an inert reversed universal CAR (Allo-RevCAR01-T) with a short-acting recombinant adapter, R-TM123, that bridges the receptor to CD123; because the engineered cells are active only while the adapter is being infused, antileukemic activity is titratable and can be switched off within hours. In the phase 1a portion of the RevSTAR-123 study (NCT05949125), 17 patients with CD123-positive relapsed/refractory or measurable residual disease–positive AML were treated: cytokine release syndrome occurred in 76% of patients (6% with grade ≥ 3), with no graft-versus-host disease or immune effector cell–associated neurotoxicity and a single grade 2 immune effector cell–associated hemophagocytic-like syndrome dose-limiting toxicity that was managed by interrupting the adapter. Response depth was dose-dependent; at the highest dose level, responses included complete remissions, measurable residual disease conversion, and a morphologic leukemia-free state in TP53-mutated AML, with a phase 1b cohort now enrolling (32). RevSTAR-123 was not restricted to posttransplant relapse, but its reversible safety switch directly addresses the on-target myelotoxicity that constrains every shared-antigen myeloid CAR described above, while its off-the-shelf, universal-donor format removes the requirements for fresh apheresis and donor recontact that limit conventional donor-derived products.

An adjacent strategy engineers the graft to lack the target antigen. Tremtelectogene empogeditemcel (trem-cel), a CRISPR-Cas9–edited CD33-deleted allogeneic stem cell product, is designed to shield the donor graft from subsequent CD33-directed therapy (33). In a multicenter phase 1/2a trial, 30 adults with high-risk AML or myelodysplastic syndromes (MDS) received trem-cel, of whom 19 went on to receive gemtuzumab ozogamicin maintenance (15 in the phase 1 dose-escalation cohort and 4 in the phase 2 dose-expansion cohort). All 30 achieved primary neutrophil engraftment; gemtuzumab ozogamicin was tolerated up to the recommended phase 2 dose of 2 mg/m² without prolonged high-grade cytopenias, demonstrating that CD33-negative donor hematopoiesis permits repeated antibody–drug conjugate dosing that would ordinarily be limited by myelotoxicity. The trial was nonetheless stopped early, and the published report is the final analysis; three transplant-related deaths occurred (renal failure, sepsis, and sinusoidal obstruction syndrome), and the most common adverse events were cytopenias and infections (33). Pairing trem-cel with a CD33-directed CAR such as VCAR33 is the intended extension. The approach remains at an early stage, and its limitations warrant acknowledgment: it is antigen-specific, since each edited graft protects against only a single target; it requires ex vivo genome editing of the graft, with attendant manufacturing complexity and scalability constraints; and it has been evaluated only in small, single-arm cohorts with limited follow-up, and randomized evidence that graft engineering plus CD33-directed maintenance reduces relapse is still awaited. Analogous CRISPR-edited CD123-deleted hematopoietic stem cell (HSC) approaches are in preclinical development (34).

Three limitations define the current role of donor-derived CAR-T: a 20% response rate that has not yet justified the operational complexity relative to autologous CAR-T or HMA–venetoclax–DLI; donor recontact and fresh apheresis requirements that exclude cord blood recipients; and the absence of a sustainable reimbursement model.

## 7 HLA-directed CAR-T and CAR-NK: exploiting donor–recipient mismatch

The autologous and donor-derived approaches share a fundamental limitation: they target myeloid antigens also expressed on normal hematopoietic progenitors, and the resulting on-target/off-tumor toxicity constrains what any construct can achieve without stem cell rescue or CRISPR-edited grafts. An alternative specific to the posttransplant setting uses the donor-recipient HLA mismatch itself as the targeting specificity. The CAR recognizes an HLA variant retained by recipient-derived leukemia but absent from donor-derived hematopoiesis; because the target is preserved on the residual patient-origin leukemic clone but not on engrafted donor hematopoietic cells, the therapy can in principle achieve leukemia specificity as an inherent property of the targeting design. Selectivity relative to non-hematopoietic tissues, which are recipient-derived and therefore retain the allele, does not depend on its genetic absence but on antigen-density- and affinity-dependent recognition, as discussed below.

The clearest realization comes from Ikeda et al. (35). The team generated thousands of monoclonal antibodies against AML cell preparations and narrowed to 32 candidates selectively binding AML over normal blood cells. Further screening identified KG2032, a monoclonal antibody binding AML blasts in approximately half of patient samples, not binding normal leukocytes (except B lymphocytes), and binding minimally to non-hematopoietic tissues even under severe inflammation. KG2032 also bound the CD34+CD38− leukemic stem cell compartment – the population responsible for posttransplant relapse.

Epitope mapping revealed that KG2032 recognizes HLA-DRB1 molecules distinguished by a polymorphism at codon 86: the antibody binds alleles in which the 86th amino acid is not aspartic acid and does not bind alleles in which it is. Approximately half of the population carries at least one KG2032-reactive HLA-DRB1 allele. Despite widespread inducibility of HLA class II on activated endothelium and inflamed parenchyma, selectivity for hematopoietic over non-hematopoietic tissue is attributed to relatively low antibody binding affinity—sufficient for recognition of high-density HLA-DRB1 on AML blasts and B lymphocytes, but insufficient for cross-reaction with lower-density induced expression elsewhere.

The therapeutic concept becomes fully leukemia-specific when the posttransplant context is incorporated. In a patient with AML carrying a KG2032-reactive HLA-DRB1 allele who received transplantation from a donor whose HLA-DRB1 alleles are both KG2032-nonreactive, engrafted donor hematopoiesis is invisible to a KG2032-directed CAR while the patient-origin leukemic clone is bound. An effector cell reintroduced into such a patient would deliver AML-restricted cytotoxicity. KG2032 CAR-T cells engineered from donor T cells lacking the reactive DRB1 allele (necessary to prevent fratricide) showed potent and specific anti-AML activity *in vitro* and in xenograft models with no overt toxicity. Cord blood–derived KG2032 CAR-NK cells showed comparable activity, extending the platform to off-the-shelf formats.

Suga and Hosen (36) positioned KG2032 within the broader HLA-directed therapeutic landscape and argued for systematic mapping of additional polymorphic class II epitopes. Eligibility of approximately 50% based on a single antibody might, in principle, be expanded toward 80–90% with a panel of three to five HLA-directed CARs covering complementary polymorphisms, which could broaden HLA-directed therapy from a narrow option toward a more widely applicable posttransplant cellular salvage class—although this remains a hypothesis pending clinical validation.

Three caveats limit current evidence. All published KG2032 data are preclinical; clinical trials were planned at publication but had not entered accrual. The platform is inherently personalized by HLA genotype; its path to availability is likely through academic consortia rather than conventional commercialization. Predictable loss of normal B lymphocytes will produce CD19-CAR-like hypogammaglobulinemia. Open questions remain regarding response durability, antigenic escape under KG2032 pressure, and the comparative performance of CAR-T versus CAR-NK. KG2032 illustrates a key point: the specificity problem of AML CAR-T may be best addressed not by finding a better single myeloid antigen, but by exploiting the immunologic differences between donor and recipient that the transplant itself has created.

## 8 CD83-directed CAR-T: dual targeting of leukemia and alloreactive T cells

A distinct approach exploits a single antigen shared between AML blasts and the alloreactive cellular effectors of GVHD. A CAR directed against that antigen can, in principle, address the two leading causes of posttransplant mortality—AML relapse and GVHD—within a single product. The antigen is CD83, currently in first-in-human evaluation.

CD83 is expressed at high surface density on AML blasts, including CD34+ primary AML cells, in which its expression exceeds that of CD33 or CD123. It is expressed on mature and pro-inflammatory dendritic cells driving alloantigen presentation in acute GVHD; is induced on alloactivated conventional CD4+ T cells while being negligibly expressed on regulatory and CD8+ T cells mediating antiviral responses; and is upregulated in B and T follicular helper cells in chronic GVHD. CD83 is absent on hematopoietic stem cells. This expression pattern identifies a single target that is simultaneously pro-leukemic, pro-GVHD, and largely absent from cell populations the transplant preserves.

The CD83-directed CAR was developed by Shrestha et al. (37)—a second-generation construct with an anti-CD83 scFv, a CD8 hinge and transmembrane domain, a 4-1BB costimulatory domain, and CD3ζ. Preclinically, CD83 CAR-T cells eradicated pathogenic CD83+ targets in xenogeneic GVHD models, increased the ratios of regulatory T cells to alloactivated conventional T cells, and killed AML cell lines and primary blasts. Critically, they preserved antiviral T-cell responses against cytomegalovirus (CMV), Epstein–Barr virus (EBV), and influenza and retained cytolytic activity in the presence of calcineurin inhibitors. Holtan et al. (38) showed that CD83 expression on circulating CD4+ T cells before day +100 correlates with increased transplant-related mortality, validating CD83 as both a biomarker and a therapeutic target.

A phase 1 trial (NCT06871410) opened at Roswell Park in early 2026 and initiated accrual (39), positioned explicitly for relapsed/refractory AML including post-HSCT patients—a niche from which most CAR-T trials exclude such patients. Clinical safety and efficacy have not been reported.

Three uncertainties remain. All activity data are preclinical. The clinical balance between GVL enhancement and GVHD attenuation in the same patient remains to be observed. Single-center manufacturing may constrain near-term availability. Nevertheless, CD83-directed CAR-T is the only cellular therapy currently in clinical evaluation explicitly designed to address both principal causes of posttransplant mortality in a single product.

## 9 CAR-NK platforms: reprogramming innate allogeneic immunity

NK cell–based CAR therapies represent fundamentally different solutions. NK cells mediate cytotoxicity through receptor-engaged and missing-self mechanisms, do not require HLA restriction, and do not carry the clonal endogenous T-cell receptor that drives GVHD in unmodified allogeneic T-cell products. A CAR-NK product manufactured from unrelated donors, cord blood, or an induced pluripotent stem cell (iPSC) master cell bank can be infused into any recipient without HLA matching, making this the most operationally scalable cellular therapy in development for post-HSCT AML relapse.

Three cell sources are clinically evaluated. Umbilical cord blood–derived NK cells are the predominant academic source—expandable to clinically relevant doses, readily modified, and carrying low GVHD risk (39). Peripheral blood NK cells from healthy donors require complex expansion protocols. iPSC-derived NK cells offer the most scalable long-term platform with more variable function. The shared constraint is limited *in vivo* persistence (two–four weeks without exogenous cytokine support), prompting IL-15 signaling cassettes and armoring strategies analogous to CAR-T (40).

The most informative published clinical experience comes from Huang et al. (42) in Experimental Hematology & Oncology: cord blood–derived NK cells modified to express a CD33-directed CAR were infused into 10 patients with R/R AML. At a median follow-up of 125 days, 6 of 10 patients (60%) achieved an MRD-negative complete response. Durability was limited: median progression-free survival was 71.5 days, median overall survival among complete responders was 137 days, and only one patient (subsequently bridged to allo-HSCT) achieved remission beyond 258 days. The safety profile was favorable. Earlier first-in-human work on CD33 CAR-NK-92 cells (43) established initial safety. Recent preclinical work by Bexte et al. (44) showed that CRISPR-Cas9 NKG2A knockout in primary CD33 CAR-NK cells substantially improves efficacy by relieving HLA-E–mediated suppression.

Cord blood–derived KG2032 CAR-NK cells produced preclinical antileukemic activity comparable to KG2032 CAR-T (35). The combination of true leukemia specificity from donor–recipient HLA-DRB1 mismatch and the off-the-shelf model afforded by cord-blood NK may represent the closest the field has come to an allogeneic CAR product without either the target-sharing problem or the operational constraints of fresh donor apheresis.

Two limitations constrain the near-term role. Limited persistence translates into durable responses in only a minority of patients, and response depth and durability have not approached benchmarks set by contemporary armored CAR-T products. Two practical questions follow. First, whether to compensate for short persistence with repeated infusions or to engineer longer persistence: repeat dosing is feasible with off-the-shelf products and is a reasonable near-term strategy, but the field is converging on persistence-enhancing engineering—IL-15 or cytokine armoring, NKG2A or CIS/CISH knockout, and CD16 enhancement, all in active development (41, 44)—as the more durable solution. Second, whether enhanced persistence introduces the risk of uncontrolled proliferation associated with some engineered T-cell products: this concern is comparatively low for NK cells, which lack a clonal integrating T-cell receptor, have finite intrinsic lifespans, and have not shown the same transformation signals, making persistence enhancement a relatively safe lever. Short persistence is moreover not disqualifying for the role CAR-NK is best suited to—transient but deep cytoreduction is exactly what a bridge to transplantation requires, and the one durable remission in the Huang cohort occurred in the patient who underwent consolidation with allo-HSCT. Persistence-enhanced CAR-NK biology combined with HLA-directed targeting may ultimately yield a product that couples specificity with operational accessibility.

## 10 A proposed clinical algorithm for 2026

The algorithm integrates the GVL Failure Framework (Section 2), salvage benchmarks (Section 4), and cellular modalities (Sections 5–9) into a single decision framework (**Figure 2**; **Table 2** contrasts platforms across selection-relevant dimensions). It is an investigational framework intended for academic centers with clinical trial access, not a validated standard-of-care guideline; several of its steps – relapse-clone 6p loss-of-heterozygosity analysis, HLA class II profiling, and KG2032 eligibility typing – are not yet routine practice at most centers. The central premise is that effective treatment of post-HSCT AML relapse is only partly a question of choosing a drug; it is primarily a question of characterizing the specific GVL failure mode and matching therapy to it.

**Table 2:** Comparison of cellular therapy platforms for post-HSCT AML relapse across the dimensions that drive clinical selection.

Dimension	Autologous CAR-T (post-HSCT)	Donor-derived CAR-T	HLA-directed CAR-T/NK (KG2032)	CD83-directed CAR-T	CAR-NK (cord blood/iPSC)
Cell source	Patient apheresis (predominantly engrafted donor T cells)	Fresh apheresis from the original HSCT donor	Donor T cells lacking reactive DRB1 allele; or cord-blood NK	Patient apheresis (donor-origin post-HSCT)	Cord blood or iPSC-derived NK; master cell bank
Target class	Shared myeloid antigen (CD33, CD371)	Shared myeloid antigen (CD33, CLL-1, CD123, CD117)	HLA-DRB1 polymorphism (donor-recipient mismatch)	Immune-cell activation marker (CD83)	Myeloid antigen or HLA (multiple constructs)
Graft-sparing by design	No (on-target myelotoxicity persists)	No (unless paired with a CRISPR-edited graft, e.g., trem-cel)	Yes – leukemia-specific by donor-recipient mismatch	Yes (CD83 absent on HSCs; sparing confirmed in CFU assays)	Construct-dependent (HLA-directed spares graft; CD33 does not)
Post-HSCT GVHD risk	Low (cells already tolerant to recipient)	Low to moderate; <10% grade ≥III in published series	Very low (donor cells lack the reactive allele; no fratricide)	Low, and may actively suppress existing GVHD	Very low (absent endogenous TCR)
Antiviral immunity	Preserved	Preserved (unless TCR-disrupted)	Preserved	Preserved (spares CD8+ and Treg compartments)	Preserved
Manufacturing model	Per-patient bespoke; lymphopenia-limited yield	Per-patient; requires donor re-contact and fresh apheresis	Personalized by HLA genotype; academic-consortium pathway	Per-patient bespoke	Off-the-shelf and scalable; suitable for multi-dose regimens
*In vivo* persistence	Moderate; host exhaustion limits expansion	Moderate; typically transient expansion (<30 days)	Uncertain (preclinical data only)	Uncertain (preclinical; early clinical)	Short (~2–4 weeks) without cytokine support
Clinical stage (mid-2026)	Phase 1 (CLEAR-AML, multiple Chinese programs)	Phase 1/2 (VCAR33 terminated; CLL-1 ongoing)	Preclinical; first-in-human trials planned	Phase 1 (Roswell Park; accrual begun)	Phase 1 (Huang CD33 CAR-NK completed)
Principal bottleneck	On-target myelotoxicity; product durability	Commercial model: cord-blood recipients excluded	HLA-genotype eligibility (~50% of mismatched pairs)	Clinical efficacy data await	Limited *in vivo* persistence
Best-fit role (Section 10 algorithm)	Cytoreductive bridge to second allo-HSCT across pathways	Bridge to second allo-HSCT; CRISPR-graft-paired strategies	Specificity-resolving salvage for eligible patients (Pathways B and C)	Post-HSCT salvage with residual GVHD concern	Scalable access for transplant-ineligible patients (Pathway D) and HLA-directed formats

Platform comparison synthesizing the mechanistic and operational features discussed in Sections 4 through 9. “Best-fit role” aligns each platform with its position in the proposed treatment algorithm (**Figure 2**, Section 10). Entries represent our interpretation of the evidence base as of mid-2026 and are expected to evolve as clinical trials mature.

A switchable, off-the-shelf allogeneic CD123 CAR-T platform (AVC-201/Allo-RevCAR01-T plus the short-acting R-TM123 adapter; RevSTAR-123) combines features of the donor-derived and CAR-NK columns—universal-donor manufacturing with adapter-controlled, reversible activity—and is discussed in Section 6. allo-HSCT, allogeneic hematopoietic stem cell transplantation; CAR, chimeric antigen receptor; CLL-1, C-type lectin-like molecule 1; CRISPR, clustered regularly interspaced short palindromic repeats; GVHD, graft-versus-host disease; HLA, human leukocyte antigen; HSC, hematopoietic stem cell; iPSC, induced pluripotent stem cell; LSC, leukemic stem cell; NK, natural killer; TCR, T cell receptor; Treg, regulatory T cell.

At the first evidence of relapse, we propose four diagnostic steps in parallel with initial cytoreduction: HLA typing of the relapsed clone (chromosome 6p copy-number analysis of sorted blasts) to determine whether same-donor DLI is biologically coherent (Category 1 detection); HLA class II expression status by flow cytometry to identify Category 2 patients most likely to benefit from hypomethylating agent exposure; a targeted molecular panel (FLT3-ITD, IDH1/2, KMT2A, NPM1, TP53); and re-interrogation of donor and recipient HLA typing for KG2032 eligibility (this fourth step does not require new samples). Copy-number/HLA-loss analysis and flow-based HLA class II assessment return quickly enough to inform pathway assignment while cytoreduction begins, whereas the targeted molecular panel, which takes longer, need not delay initial treatment or pathway selection.

Pathway A (late >12-month MRD-only relapse, HLA-intact clone, favorable molecular profile) begins with rapid taper of immunosuppression, azacitidine–venetoclax if tolerated, and planned low-dose DLI if MRD recurs; mutation-specific oral agents are added where applicable. Most Pathway A patients do not require cellular engineering.

Pathway B (early or morphological relapse, candidate for second allo-HSCT) prioritizes cytoreduction with azacitidine–venetoclax (intensive chemotherapy for proliferative disease). Once CR2 with MRD-negativity is achieved, second transplantation proceeds, ideally from an alternative donor when HLA loss is documented. Cellular salvage enters only if conventional cytoreduction cannot achieve CR2: KG2032 CAR-T/NK if mismatch criteria are met; CD83 CAR-T for residual GVHD concerns; armored CD371/IL-18 for CD371-positive disease; CD33-directed CAR-T or CAR-NK as the most broadly accessible fallback. Current evidence positions these as bridges to transplantation.

Pathway C (Category 1 antigen-loss relapse) requires explicit separation. Same-donor DLI is unlikely to produce meaningful GVL and is generally not recommended unless preserved donor-recognizable HLA epitopes are documented. If the patient is a second-transplant candidate, the preferred donor differs from the first at loci other than the lost haplotype. HLA-directed cellular therapy targeting a non-lost locus (e.g., KG2032 when HLA-DRB1 is preserved) can be applied if an informative mismatch remains. Hypomethylation retains utility for class II re-expression independent of HLA loss status. Pathway C intersects the clinical eligibility axis rather than replacing it: a patient with antigen-loss relapse who is also transplant-ineligible should follow the Pathway D principles below while still avoiding biologically incoherent same-donor DLI.

Pathway D (transplant-ineligible, per the clinical eligibility axis) is where novel cellular therapies may eventually have the greatest incremental impact. The current standard is azacitidine–venetoclax with or without low-dose DLI, supplemented by molecularly targeted therapy and palliative integration. Enrollment in CAR-T or CAR-NK trials should be actively pursued, particularly for armored or HLA-directed constructs that may not require subsequent transplantation (Section 11).

Three sequencing principles apply. Apheresis for autologous CAR-T should be performed as early as possible. Donor search and second-transplant planning should proceed in parallel with cellular therapy. HLA class II and HLA-loss status on the relapse clone should inform therapeutic decisions upfront.

## 11 Beyond bridging: can cellular therapy replace second transplantation?

The cellular therapies described are currently positioned as cytoreductive bridges to second allogeneic transplantation. Durable remissions are largely confined to patients who underwent consolidation with a second transplant (25, 26, 28). A different question is whether the maturing platforms—particularly armored and HLA-directed constructs—may eventually replace second transplantation rather than bridge to it.

Three observations argue this question is worth asking now. First, second allo-HSCT carries substantial non-relapse mortality that has not improved across recent eras even as relapse rates have fallen (23); the procedure remains intensive enough to exclude most comorbid or transplant-toxicity-burdened patients. Second, sustained MRD-negative remissions in the CLEAR-AML CD371/IL-18 cohort demonstrate that armored constructs can produce rapid disease clearance with single-agent activity (25). Third, the leukemia-restricted specificity afforded by HLA-directed approaches such as KG2032 (35) creates the possibility of cellular therapy that does not damage the engrafted graft and therefore does not require a second graft to rescue hematopoiesis.

Four criteria would need to be met before transplant-sparing cellular therapy enters serious consideration: durability of remission reaching 2–3 years (current data extend to ~12 months); characterization of antigenic escape under HLA-directed pressure with mitigation through dual targeting or expanded panels (36); acceptable GVHD-related toxicities without subsequent transplant rescue; and prospective quality-of-life and cost comparisons.

Durability will also depend on anticipating how leukemia escapes not only the original graft but also the new platforms. The same escape biology catalogued in the GVL Failure Framework is likely to recur under cellular-therapy pressure: an HLA-directed CAR such as KG2032 may select for epigenetic silencing or genomic loss of the targeted HLA-DRB1 allele, recapitulating the antigen-silencing and antigen-loss mechanisms that drive relapse after conventional transplantation, while single-antigen myeloid CARs against CD33 or CD123 remain vulnerable to antigen-low escape and myeloid-to-lymphoid lineage switching. These considerations provide the central argument for dual- and multi-antigen constructs (for example, CD33–CLL-1) and for expanded HLA-directed panels: redundancy in targeting is what would convert transient cytoreduction into the transplant-free durability envisioned in this section.

The clearest near-term test population is Pathway D—patients ineligible for second transplantation. For this group, cellular therapy competes against palliation, not transplantation. A signal of durable benefit here would justify dedicated trials of transplant-sparing cellular therapy in fit candidates. Until such evidence accumulates, the prudent position is cellular therapy as a cytoreductive bridge in transplant-eligible patients and as definitive therapy only within clinical trials for those with no further transplant option.

Two non-CAR modalities pursue the same goal of redirected, leukemia-specific immunity and fall outside this review’s focus on chimeric antigen receptor engineering, but warrant brief mention. T-cell receptor-engineered T cells (TCR-T cells) redirect T cells against intracellular leukemia-associated antigens or minor histocompatibility antigens rather than surface targets: donor-derived WT1-specific TCR-T cells administered after transplantation were associated with 100% relapse-free survival at a median of 44 months in high-risk AML (45), and HA-1–directed TCR-T cells—exploiting a recipient-restricted minor histocompatibility antigen analogous in logic to the HLA-mismatch strategy described in Section 7—proved feasible and tolerable for recurrent leukemia after transplantation, with early signals of activity (46); PRAME is a further antigen under TCR-T development. Separately, CD3-engaging bispecific antibodies, including CD123×CD3 constructs such as flotetuzumab and vibecotamab, redirect endogenous T cells without genetic engineering or manufacturing delay and have shown salvage activity in refractory AML (47). These platforms are complementary to, rather than competitive with, the cellular approaches reviewed here, and several share the donor–recipient and antigen-density principles central to the GVL Failure Framework; a dedicated comparison is beyond the present scope.

## 12 Discussion

AML relapse after allogeneic transplantation remains the dominant cause of posttransplant mortality, and conventional salvage benefits only a minority of patients. The cellular therapies described here offer rational, biology-driven responses, with each modality positioned to address a specific category of GVL failure. Three features distinguish the framework proposed in this review: it organizes contemporary cellular therapy around the underlying biology of immune escape rather than treatment class; it elevates relapse-clone HLA characterization as a prerequisite for biologically coherent treatment selection—particularly before DLI; and it separates patients in whom HLA-directed and CD83-directed approaches are biologically rational from those in whom they are not. Significant gaps remain. The therapies with the strongest conceptual arguments—KG2032 and CD83-directed CAR-T—have the least mature clinical data, head-to-head comparisons are absent, and the rare-disease economics of posttransplant relapse suggest academic consortia will carry much of the development burden. Antigenic escape under cellular therapy pressure remains a concern that dual-targeting and expanded HLA-directed panel approaches must address. An international registry stratified by relapse biology, together with multinational consortium infrastructure, would accelerate trial accrual and align development with the global epidemiology of AML and allo-HSCT. Until evidence matures, aligning practice with biology—characterizing the failure mode and matching the therapy—offers the clearest path to improving outcomes in this population. The future challenge in posttransplant AML relapse is not merely developing new cytotoxic therapies, but identifying the specific mode of GVL failure and selecting cellular platforms capable of restoring immune control.
